# Evaluating Intra-household Agreement on Multi-domain Family-Level Social Determinants of Health and Exploring Individual Correlates of Agreement in Two Southern California Family Studies

**DOI:** 10.1007/s11121-026-01914-2

**Published:** 2026-05-12

**Authors:** Alexandra Descarpentrie, Payal Shah, Sevan Esaian, Kerri N. Boutelle, Dawn M. Eichen, Tanya L. Alderete, Michael I. Goran, Juan Espinoza

**Affiliations:** 1 Department of Pediatrics, Children’s Hospital Los Angeles, University of Southern California, Los Angeles, CA USA; 2 Department of Pediatrics, Herbert Wertheim School of Public Health and Longevity Science and Psychiatry, University of California, San Diego, CA USA; 3 Department of Pediatrics, University of California San Diego, La Jolla, San Diego, CA USA; 4 Department of Environmental Health and Engineering, Johns Hopkins Bloomberg School of Public Health, Baltimore, MD USA; 5 Stanley Manne Children’s Research Institute, Ann & Robert H. Lurie Children’s Hospital of Chicago, Chicago, IL USA; 6 Department of Pediatrics, Northwestern University Feinberg School of Medicine, Chicago, IL USA

**Keywords:** Intra-household agreement, Family-level social determinant of health, Individual-level correlates, Latino, Hispanic, California

## Abstract

Accurate self-reported data on social determinants of health (SDoH) are essential for improving prevention initiatives. Beyond survey content and validation, deciding which household member should complete family-level SDoH assessments can affect data quality. Yet, few studies have explored how family members report on various SDoH, especially in the Latino/Hispanic community experiencing greater health challenges. This study not only examined intra-household agreement on family-level SDoH items but also assessed combined individual SDoH linked to agreement in Southern California, which hosts one of the largest Latino/Hispanic communities in the USA. We analyzed data from 277 adult pairs (*n* = 554) across Southern California who completed the National Institute on Minority Health and Health Disparities Common Data Elements questionnaire. Each respondent answered 19 household-level items across four domains: *Demographics*, *Economics*, *Health and Clinical Care*, and *Housing*. Agreement was evaluated using simple or weighted Cohen’s Kappa (range, ≤ 0 to 1). Modified Poisson regression examined associations between agreement and combined individuals’ health literacy, employment, sex at birth, age, birthplace, and ethnicity. Tests were corrected for multiple comparisons. Among respondents, 56.3% were women, participants’ mean age was 41.8 years (standard deviation = 8.6), 93.1% identified as Latino/Hispanic, and 37% reported low health literacy. Agreement across domains varied (Kappa = 0.14–0.85), with higher agreement observed for *Demographics* and transportation items, and poor agreement on financial adversity, healthcare, and food insecurity items. Agreement on food insecurity items varied by dyad composition: same-sex dyads demonstrated greater agreement than mixed-sex dyads, whereas dyads in which both members identified as Latino/Hispanic showed lower levels of agreement. Variations seem to exist in how household members report on family-level SDoH, particularly for sensitive areas like finances and food access. To improve accuracy, researchers and public health professionals might consider collecting data from multiple household members while encouraging joint responses and accounting for specific when designing assessments.

**Graphical Abstract:**

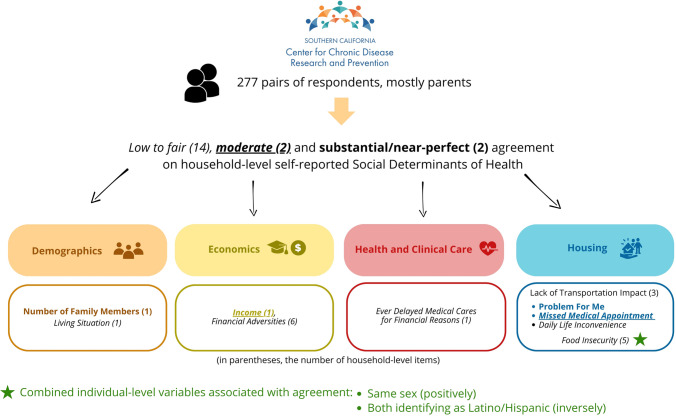

**Supplementary Information:**

The online version contains supplementary material available at 10.1007/s11121-026-01914-2.

## Introduction

For decades, increases in chronic diseases (e.g., obesity, type 2 diabetes) and related persistent health disparities in the USA have been well-documented (Zenk et al., 2024). To try to address these disparities, social determinants of health (SDoH), or “the conditions in which people are born, grow, work, live, and age, along with the systems and forces that shape daily life,” are increasingly being recognized as a priority by stakeholders (Office of Disease Prevention & Health Promotion, 2022). SDoH encompass a broad and multifaceted set of health influences that span multiple domains, the conceptualization of which varies across frameworks (Chen et al., 2025; Tanarsuwongkul et al., 2025). A recognized framework, articulated by Healthy People 2030, delineates five domains: economic stability, education access and quality, healthcare access and quality, neighborhood and built environment, and social and community context (US Department of Health and Human Services, n.d.). From a public health perspective, efforts to address these SDoH are crucial in Southern California, where the Latino/Hispanic community (one of the largest in the USA; comprising 45% of the regional population compared with 20% nationally) faces heightened health challenges, including conditions such as obesity and diabetes, alongside socio-economic resource limitations (Batista & Gonzalez-Guarda, 2025; Cleveland et al., 2023).

Effectively targeting these health-related SDoH first requires careful attention not only to survey content (such as question selection, framing, and response options) and validation, but also to how data are collected. Individual-level SDoH, which capture the circumstances and resources specific to a single person, are relatively straightforward to collect. Beyond the individual, group-level SDoH (encompassing family, household, neighborhood, or community conditions) reflect the broader social context in which people live (Diez-Roux, 1998). Focusing on household- or family-level SDoH is particularly important because many health-related conditions (such as income, housing stability, food security, and access to transportation) are shared across household members and often organized at the household level. Considering the social and economic realities of adult household members provides valuable insight into the broader context in which the health and well-being of all members are shaped. However, relying on a single adult respondent, while more practical and cost-effective, may offer only a partial view of a potentially complex household dynamic (Masselus & Fiala, 2024). For example, a study in Minneapolis/St. Paul found that Latino mothers reported food insecurity at a higher rate (55%) compared with fathers (39%) (Nagao-Sato et al., 2021), a disparity attributed to differences in individuals’ participation in nutrition education and perception of accessibility of fruits and vegetables at home. Only a limited number of studies have examined agreement across a wide spectrum of family-level SDoH and explored how the individual characteristics of household members shape that agreement, particularly within Latino/Hispanic communities in Southern California. Understanding where and why discordance occurs can reveal which SDoH are reliably reported and ultimately strengthen both research accuracy and thereafter interventions tailored to household needs.

Within Latino/Hispanic communities in Southern California, the objectives of our study were therefore to (1) evaluate intra-household agreement family-level SDoH items, focusing on adult dyads and key variables from various domains known to influence health outcomes (Chen et al., 2025; Nagao-Sato et al., 2021; Tanarsuwongkul et al., 2025), and (2) identify correlates of agreement (evaluated in aim 1) based on combined individual characteristics. We hypothesized that intra-household agreement on multi-domain SDoH perceptions would vary, with greater consensus on more objective factors (e.g., demographics) and lower concordance for more perception-based or experience-dependent domains (e.g., financial adversities or food insecurity) (Nagao-Sato et al., 2021). We also hypothesized that household members with similar individual socio-demographic characteristics (e.g., employment status, sex) would exhibit higher agreement in their perceptions of family-level SDoH, consistent with literature suggesting that shared roles, responsibilities, and lived experiences may influence perceptions and reporting of circumstances (Nutbeam, 2008; Vigil, 2021).

## Methods

### Study Setting

We leveraged data from two research projects (outlined below) within the Southern California Center for Chronic Disease Research and Prevention (SC3DRP), each collecting household- and individual-level SDoH information from multiple family members (the present analysis focuses on a single adult dyad per household). Briefly, the SC3DRP is a National Institute on Minority Health and Health Disparities (NIMHD)–funded research center based at Children’s Hospital Los Angeles focused on understanding why certain chronic diseases (especially, obesity, type 2 diabetes, non-alcoholic fatty liver disease, and dyslipidemia) disproportionately impact Latinos/Hispanics. The center serves as a regional hub across ten counties in Southern California and, among other activities, hosts four R01-like projects that explore different aspects of health disparities. Among these projects, common data elements (CDEs) are consistently collected, enabling the integration of data across studies.

#### The Family, Responsibility, Education, Support, and Health for Latino Caregivers (FRESH-LC) Study

The FRESH-LC study (NCT05437406) is a 6-month, no-cost telehealth weight management intervention culturally tailored for Latino/Hispanic families with a child who has overweight or obesity, conducted by the University of California, San Diego. Both a parent and an additional caregiver, living in San Diego or the surrounding areas, participate in the treatment, with all participation conducted remotely in both Spanish and English. The FRESH-LC study (recruitment started on September 2022 and ended February 2025) is a culturally tailored parent-based treatment for childhood obesity, focusing on feeding behaviors, eating, and physical activity to promote weight loss. Across the 167 households in the final sample, 134 comprised adult dyads sharing the same residence and were therefore considered for this analysis.

#### The Food Prescriptions to Promote Affordable Diets that Meet RDAs Among Multi-Generational Latino Households (FoodRx) Study

The FoodRx study (NCT05309395, recruitment started August 2021 and ended June 2025) is an affordable and culturally sensitive meal-planning and grocery delivery intervention for Latino/Hispanic multi-generational households from Kaiser Permanente members in the Los Angeles metro area. Its goal is to assess the impact of food prescriptions and grocery delivery on parental and child obesity, diet quality, and chronic disease risk. A subsample of 143 households, all sharing the same residence, was selected for this analysis, according to the data available at the time of the analysis (May 2025). Where more than two adults could be included in a household, we selected the first two adult respondents based on their identifiers to maintain consistency in the selection of dyads across the study.

### Self-reported Social Determinants of Health (SDoH) Data Collection

**Fig. 1:**
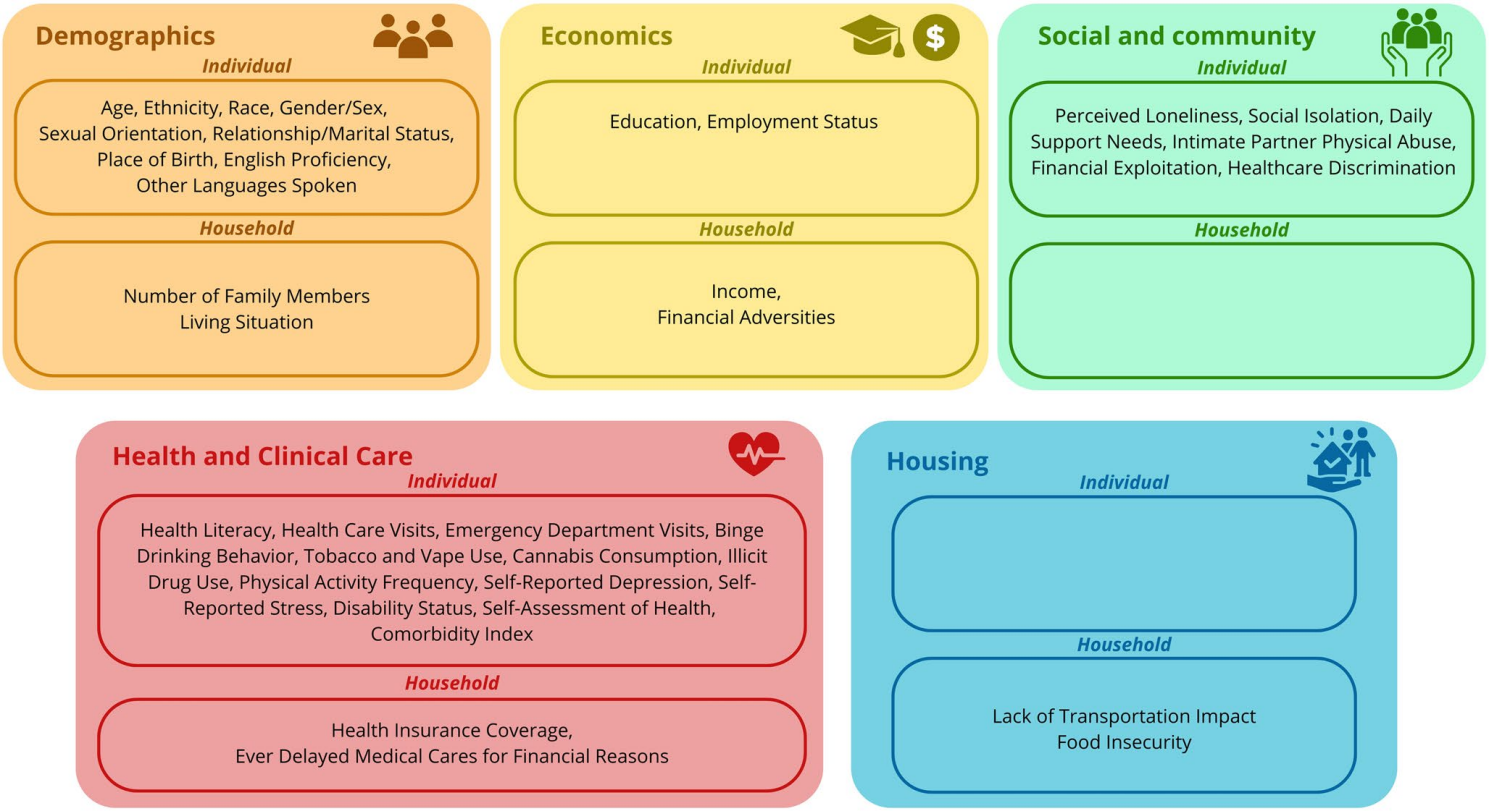
Common data elements mapping

The Multiple Chronic Diseases Disparities Research Consortium (MCD-DRC), comprising 11 research centers across the USA, including the SC3DRP, was funded by NIMHD in 2021 (https://health-excellence-action.org/). The consortium aims to advance health equity in the prevention, treatment, and management of multiple chronic diseases such as diabetes, obesity, hypertension, coronary heart disease, congestive heart failure, chronic kidney disease, stroke, and certain cancers. This consortium developed a set of CDEs to be used as a standardized set of study questions to ensure consistency in data collection and analysis across centers and research projects. A standardized REDCap survey was developed iteratively with input from all participating centers and leveraging validated items from the PhenX Toolkit and other sources that cover key areas such as SDoH, comorbidities, and quality of life (https://health-excellence-action.org/nih-cde). At baseline, each study collects up to 56 CDEs, which are categorized into two levels: household and individual. Inspired by previously developed SDoH frameworks (Bronfenbrenner, 1981; US Department of Health and Human Services, n.d.), we created a mapping of these elements provided in Fig. 1.

For our analysis, based on the literature and available data, we focused on evaluating the agreement between pairs of respondents for 19 self-reported household-level SDoH CDEs (Chen et al., 2025; Nagao-Sato et al., 2021; Tanarsuwongkul et al., 2025). Additional details about the selected items are available in Supplemental Material 1. Drawing on the available CDEs and supported by the literature (Smith et al., 2020), the combined individual-level SDoH included in the correlate analysis were as follows: excellent health literacy operationalized as both respondents reporting “extremely confident” on a single-item question from the BRIEF health literacy screening tool (*How confident are you filling out medical forms by yourself?*, 5-point Likert scale: extremely, quite a bit, somewhat, a little bit, not at all), versus at least one respondent choosing a lower category (Chew et al., 2004); employment status (both working versus at least one not working); sex at birth (both the same sex versus at least one different sex); age (both individuals falling on the same side of the sample median (40 years), either below or above, versus at least one in a different category: approach chosen for interpretability and statistical stability); birthplace (both born outside the USA versus at least one born in the USA); and ethnicity (both identifying as Latino/Hispanic versus at least one not).

### Analyses

#### Assessing Intra-household Agreement on Family-Level SDoH Items

We calculated interrater agreement for each of the 19 household-level SDoH items individually using Cohen’s Kappa, resulting in 19 separate Kappa coefficients. It is worth noting that unanswered items were not excluded; instead, non-responses were retained and coded as not available (NA), comprising approximately 0–5% of item-level data. The simple Kappa metric was used for nominal items to assess agreement beyond what would be expected by chance (Gisev et al., 2013; Warrens, 2015). Where appropriate, we also used the weighted Kappa index with equal weights to evaluate chance-corrected dyad agreement for the ordinal items (i.e., number of family members, income, and two items from the household insecurity questionnaire—Supplemental Material 1). Kappa values were evaluated as follows: ≤ 0 = no agreement; 0.01–0.20 = none to slight (low) agreement; 0.21–0.40 = fair agreement; 0.41–0.60 = moderate agreement; 0.61–0.80 = substantial agreement; and 0.81–1.00 = close to perfect agreement (Warrens, 2015).

#### Estimating Individual-Level Correlates of Agreement

For each dyad and household-level SDoH, we created binary variables coded 1 if both respondents classified the household in the same category, and 0 otherwise. Specifying these agreement variables as the outcomes in multi-variable modified Poisson regression models (Zou, 2004), we estimated the extent to which the combined individual-level characteristics mentioned above (health literacy, employment status, age, sex, birthplace, ethnicity) explained agreement across domains. Modified Poisson models are preferred for estimating risk or prevalence ratios, particularly when the model may be susceptible to misspecification (Chen et al., 2018). In our study, due to the cross-sectional design, prevalence ratios (PRs) were reported as the appropriate measure of association. Models included all combined individual-level factors and were further adjusted for the study variable (FRESH-LC, FoodRx).

#### Sensitivity Analyses

Although living together was a prerequisite for inclusion in this analysis (see “Study Setting” section), some household members nevertheless reported living alone in the “Current Living Situation” CDE item (Table 1). We thus conducted a sensitivity analysis restricting the sample to dyads in which both respondents indicated not living alone on this same CDE item (*n* = 174 households). This allowed us to assess whether agreement patterns differed meaningfully from the main (unfiltered) analysis and to determine whether any observed disagreement stemmed from imperfect sample selection or differing interpretations of the question. Intra-agreement analyses were also conducted separately for the FRESH-LC and FoodRx studies to assess potential variations in agreement patterns, given the unique social characteristics of each study population (Supplemental Materials 2 and 3).

Analyses were conducted in R (version 4.3.3). For each objective, we reported both unadjusted and Benjamini-Hochberg–corrected *p*-values to account for multiple comparisons, and interpretations were based on statistically significant corrected *p*-values.

## Results

### Participant Characteristics

The majority of respondents were parents (around 90%), with a smaller number being grandparents and uncles/aunts. Most respondents were female, comprising 56.3% of the sample (312 out of 554), with a high percentage identifying as Latino/Hispanic (93.1%) (Table 1). The sample had a mean age of 41.8 years (standard deviation = 8.6), with ages spanning from 18.0 to 75.3 years. Around 37% of respondents reported low health literacy, while 17.9% were unemployed at the time of the survey. Around 40% of respondent pairs both had good health literacy, 68.4% were both employed, 88.8% both identified as Latino/Hispanic, 25.2% were both born outside the USA, and 14.5% shared the same sex. Among those who shared the same sex, most were mother–grandmother, father–grandfather, or uncle–father pairs. Overall, around 21.5% of respondents reported living alone. Descriptive statistics by study are provided in Supplemental Materials 2 and 3. Briefly, the FRESH-LC study included more respondent pairs who had low health literacy, were unemployed, were single, earned less per month, and experienced more financial adversities on average compared to the FoodRx study.

**Table 1:** Distribution of Individual- and Household-Level Social Determinants of Health According to the Respondent Considered (*n* = 277 households)

	Respondent 1 (n* = 277)	Respondent 2 (n* = 277)
	Frequency (%) or mean (SD)	Frequency (%) or mean (SD)
Individual level		
Demographics		
Sex (female)	226 (81.6)	86 (31)
Ethnicity (Latino/Hispanic)	269 (97.1)	247 (89.2)
Age (years)	41.2 (6.59)	42.4 (10.3)
Birthplace (outside the USA)	109 (39.4)	117 (42.2)
Economics		
Employment status (unemployed)	52 (18.8)	47 (17)
Health literacy (not excellent)	73 (26.4)	132 (47.6)
Household level		
Demographics		
Number of family members (3)	70 (25.3)	65 (23.5)
Living situation (alone)	74 (26.7)	45 (16.2)
Economics		
Income (> more than $103.269/per year)	96 (34.7)	94 (33.9)
Financial adversities		
Trouble paying medical (yes)	15 (5.4)	14 (5)
Trouble paying food (yes)	24 (8.7)	19 (6.9)
Trouble paying electricity (yes)	33 (11.9)	22 (7.9)
Trouble paying housing (yes)	32 (11.6)	29 (10.5)
Trouble paying transport (yes)	13 (4.7)	16 (5.8)
Trouble paying debts (yes)	68 (24.6)	50 (18)
Trouble paying childcare (yes)	14 (5)	6 (2.2)
Health and medical care		
Ever delayed medical care for financial reasons (yes)	37 (13.4)	31 (11.2)
Housing		
Lack of transportation impact		
Missed medical appointment (yes)	3 (1.1)	5 (1.8)
Daily life inconvenience (yes)	8 (2.9)	9 (3.2)
Problem for me (yes)	132 (47.6)	128 (46.2)
Food insecurity		
Cut size or skip meals (yes)	19 (6.9)	17 (6.1)
Eat less than you felt (yes)	9 (3.2)	10 (3.6)
Could not afford balanced meal (often true)	13 (4.7)	7 (2.5)
No money to get more food (often true)	4 (1.4)	4 (1.4)
Hungry but did not eat (yes)	9 (3.2)	14 (5)

*n max; *SD* standard deviation

### Assessing Intra-household Agreement on SDoH Items

**Fig. 2:**
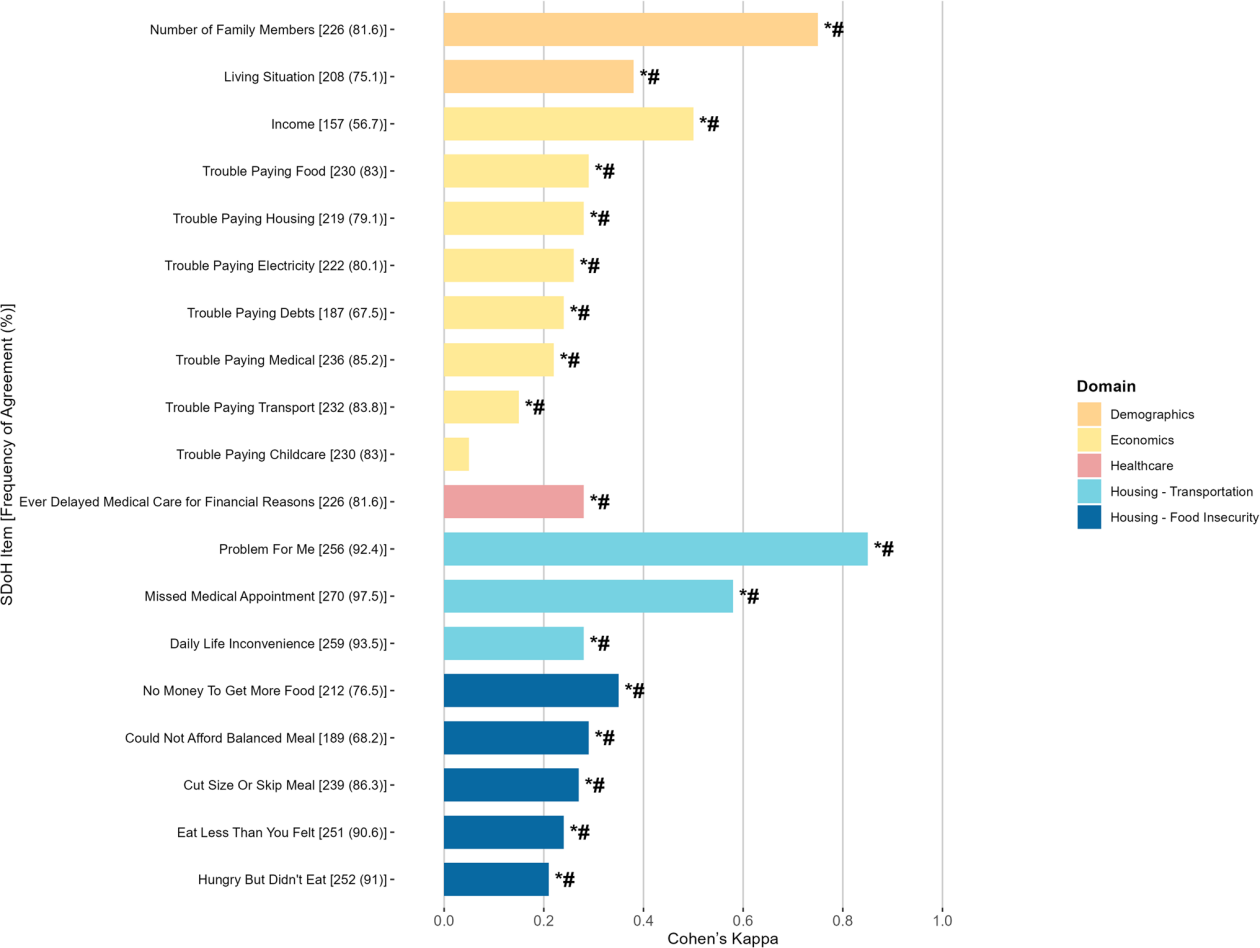
Self-reported household social determinants of health agreement assessment (*n* = 277 households). For each (sub)domain, variables are displayed in decreasing order of Cohen’s Kappa. Cohen’s Kappa goes beyond simply calculating the percentage of items agreed upon by raters; it accounts for the possibility of chance agreement. Its values range from 0 to 1, with 0 indicating no agreement and 1 indicating perfect agreement between the raters. Cohen’s Kappa can also be negative (disagreement). *p < 0.05 (Kappa test, non-adjusted *p*-values); #*p *< 0.05 (after adjusting for multiple comparisons). *SDoH* social determinants of health

In terms of *Demographics*, as displayed in Fig. 2, substantial agreement was observed on the “Number of Family Members” (Kappa = 0.75), while “Living Situation” showed fair agreement (0.37). Within the *Economics* domain, only “Income” had moderate agreement (0.51); all other financial adversity items ranged from low to fair agreement (0.14–0.28). Agreement was also fair for the item on delayed medical care due to cost (0.28). For the transportation sub-domain (*Housing* domain), the “Problem for Me” indicator showed near-perfect agreement (0.85), with moderate agreement on “Missed Medical Appointments” (0.58), and fair agreement on “Daily Life Inconvenience” (0.28). For the food insecurity sub-domain, all five indicators showed fair agreement (0.21–0.35).

### Estimating Individual-Level Correlates of Agreement

Higher health literacy among both respondents was associated with greater agreement on two items within the *Demographics* and *Economic* domains (Table 2): “Living Situation” (PR = 1.19; 95% CI, 1.01–1.39) and “Trouble Paying Electricity” (PR = 1.13; 95% CI, 1.01–1.27). Similarly, employment was associated with higher agreement on two transportation-related items: “Problem for Me” (PR = 1.15; 95% CI, 1.03–1.28) and “Daily Life Inconvenience” (PR = 1.14; 95% CI, 1.04–1.26). However, these associations did not remain significant after correction for multiple comparisons. Dyads in which both members shared the same sex demonstrated greater agreement on the food insecurity item “Could Not Afford a Balanced Meal” (PR = 1.42; 95% CI, 1.19–1.71), which remained significant after correction. In contrast, these dyads showed lower agreement on “Ever Delayed Medical Care for Financial Reasons” (PR = 0.72; 95% CI, 0.53–0.97), although this association was no longer significant after correction. Agreement on food insecurity items was also lower among dyads both identifying as Latino/Hispanic, including “Eat Less Than You Felt You Should” (PR = 0.89; 95% CI, 0.84–0.94; also significant after correction), “Could Not Afford a Balanced Meal” (PR = 0.83; 95% CI, 0.70–0.98; non-significant after correction), and “Hungry But Didn’t Eat” (PR = 0.90; 95% CI, 0.85–0.95; also significant after correction). No associations were observed for combined age or birthplace.

**Table 2:** Combined individual-level correlates of agreement on self-reported social determinants of health (*n* = 250** households)

	Excellent health literacy (both respondents) PR (CI 95%)	Employed (both respondents) PR (CI 95%)	Same sex (both respondents) PR (CI 95%)	Same age category^a^ (both respondents) PR (CI 95%)	Born outside the USA (both respondents) PR (CI 95%)	Latino/Hispanic (both respondents) PR (CI 95%)
Outcomes (agreement on …)						
Demographics						
Number of family members	1.13 (1.00; 1.29)	0.99 (0.85; 1.15)	0.80 (0.63; 1.03)	0.92 (0.81; 1.04)	1.02 (0.86; 1.20)	0.92 (0.79; 1.07)
Living situation	1.19 (1.01; 1.39)*	1.00 (0.83; 1.20)	0.90 (0.68; 1.20)	1.04 (0.87; 1.23)	1.02 (0.84; 1.24)	1.00 (0.80; 1.26)
Economics						
Income	1.23 (0.98; 1.54)	0.88 (0.69; 1.13)	0.87 (0.6; 1.25)	0.94 (0.74; 1.2)	0.88 (0.64; 1.22)	1.20 (0.84; 1.73)
Financial adversities						
Trouble paying medical	1.09 (1.00; 1.18)	1.10 (0.97; 1.25)	0.89 (0.74; 1.09)	0.97 (0.87; 1.08)	0.95 (0.83; 1.10)	0.99 (0.86; 1.14)
Trouble paying food	1.10 (0.99; 1.21)	1.10 (0.95; 1.27)	0.98 (0.79; 1.20)	1.00 (0.87; 1.14)	0.93 (0.79; 1.08)	0.97 (0.84; 1.11)
Trouble paying electricity	1.13 (1.01; 1.27)*	1.15 (0.97; 1.37)	1.01 (0.81; 1.25)	1.02 (0.88; 1.17)	0.89 (0.74; 1.08)	0.99 (0.83; 1.17)
Trouble paying housing	1.10 (0.98; 1.24)	1.16 (0.98; 1.38)	0.95 (0.75; 1.19)	0.97 (0.85; 1.12)	0.87 (0.72; 1.06)	0.99 (0.83; 1.17)
Trouble paying transport	1.09 (0.98; 1.21)	1.14 (0.98; 1.31)	0.96 (0.79; 1.17)	0.98 (0.86; 1.11)	1.02 (0.89; 1.18)	0.99 (0.85; 1.16)
Trouble paying debts	1.13 (0.95; 1.34)	1.21 (0.96; 1.53)	0.83 (0.59; 1.16)	0.95 (0.79; 1.15)	1.01 (0.80; 1.27)	0.99 (0.77; 1.28)
Trouble paying childcare	1.10 (0.99; 1.23)	1.05 (0.92; 1.19)	0.83 (0.65; 1.04)	0.99 (0.88; 1.12)	1.05 (0.92; 1.20)	1.04 (0.86; 1.25)
Health and medical care						
Ever delay medical care for financial reasons	1.00 (0.89; 1.12)	1.05 (0.91; 1.21)	0.72 (0.53; 0.97)*	1.08 (0.93; 1.26)	0.90 (0.76; 1.08)	0.92 (0.78; 1.08)
Housing						
Lack of transportation impact						
Missed medical appointment	1.00 (0.98; 1.03)	1.03 (0.98; 1.08)	0.97 (0.89; 1.06)	1.00 (0.95; 1.05)	0.99 (0.93; 1.05)	0.98 (0.95; 1.01)
Daily life inconvenience	1.02 (0.96; 1.08)	1.14 (1.04; 1.26)*	0.98 (0.86; 1.12)	1.00 (0.93; 1.09)	1.01 (0.92; 1.10)	0.98 (0.90; 1.06)
Problem for me	1.05 (0.98; 1.13)	1.15 (1.03; 1.28)*	0.97 (0.84; 1.12)	1.00 (0.92; 1.09)	1.02 (0.93; 1.12)	0.97 (0.89; 1.05)
Food insecurity						
Cut size or skip meals	1.06 (0.97; 1.16)	1.04 (0.93; 1.17)	1.00 (0.86; 1.18)	1.06 (0.94; 1.20)	0.97 (0.85; 1.10)	0.90 (0.82; 1.00)
Eat less than you felt	1.01 (0.94; 1.10)	1.06 (0.97; 1.17)	0.99 (0.86; 1.14)	1.08 (0.97; 1.20)	1.04 (0.94; 1.14)	0.89 (0.84; 0.94)*#
Could not afford balanced meal	1.14 (0.96; 1.34)	1.06 (0.88; 1.27)	1.42 (1.19; 1.71)*#	1.18 (0.97; 1.43)	0.79 (0.61; 1.01)	0.83 (0.70; 0.98)*
No money to get more food	1.00 (0.87; 1.15)	1.00 (0.86; 1.16)	0.94 (0.76; 1.17)	1.14 (0.96; 1.34)	0.90 (0.75; 1.08)	0.89 (0.76; 1.04)
Hungry but did not eat	1.07 (0.99; 1.16)	1.07 (0.97; 1.18)	0.97 (0.84; 1.12)	1.01 (0.92; 1.11)	1.08 (0.99; 1.19)	0.90 (0.85; 0.95)*#

Models were all adjusted for all the individual-level correlates and the study variable (FRESH-LC, FoodRx)

*CI* confidence interval, *PR* prevalence ratio (from multi-variable models)

*p < 0.05 (non-adjusted *p*-values); #*p* < 0.05 (after adjusting for multiple comparisons)

**To ensure analytical consistency, households with incomplete information on individual-level SDoH were excluded from the analysis. This exclusion applied to 27 households

^a^Below or above the median age (40 years old)

### Assessing Intra-household Agreement on SDoH Items: Using Only Dyads that Jointly Self-declared Not Living Alone

When considering only households in which both respondents reported living with others, the agreement patterns were similar (Supplemental Material 4) to those in the main analysis (Fig. 2).

### Assessing Intra-household Agreement on SDoH Items: The FRESH-LC vs. FoodRx Study

In the FRESH-LC study, which included participants with few social resources (Supplemental Material 2), agreement among respondents was generally low to fair, especially in the *Economic* domain and areas of financial adversities, with Kappa values ranging from − 0.05 to 0.24 (Supplemental Material 5). In contrast, respondents from the FoodRx study showed higher agreement for these items, with Kappa values ranging from 0.16 to 0.48 (Supplemental Material 6).

## Discussion

Our study explored the agreement between pairs of household members’ reports of family-level SDoH in Southern California and found lower agreement in some domains, though better agreement was observed for demographic characteristics and the impact of transportation issues. Agreement on food insecurity items varied by dyad composition: same-sex dyads demonstrated greater agreement than mixed-sex dyads, whereas dyads in which both members identified as Latino/Hispanic showed lower levels of agreement.

### Comparison with Other Studies

As observed in earlier studies focusing on individual SDoH measures, our findings showed low to moderate intra-household agreement, particularly among parent dyads. For instance, Nagao-Sato et al. found that mother–father pairs in Minneapolis disagreed on their household’s food security status (Nagao-Sato et al., 2021), while Smith et al. demonstrated that 43% of rural Ugandan households would fall into different wealth quintiles depending on whether the husband or wife completed the survey (Smith et al., 2020). Our study extends these findings by examining multiple SDoH dimensions rather than isolated indicators. Notably, we found the strongest agreement in the *Demographics* domain and transportation sub-domain. This higher agreement is unsurprising given the objective nature of these items; however, even in these domains, only moderate agreement was achieved, highlighting persistent discrepancies in self-reporting.

Dyads consisting of respondents of the same sex showed higher agreement on the “Could Not Afford a Balanced Meal” food insecurity item. This may be attributed to shared experiences, communication styles, or cultural expectations that influence how individuals interpret and report SDoH (Vigil, 2021). Finally, aligning with our findings, prior research has documented discrepancies in food insecurity reporting between Latino mothers and fathers (speaking Spanish). Nagao-Sato et al. attributed much of this discordance to dyadic differences, particularly in nutrition education participation and perceived accessibility of fruits and vegetables within the household (Nagao-Sato et al., 2021). The authors proposed that offering nutrition education to fathers could help mitigate reporting discordance within dyads by enhancing their awareness of household food availability and shaping perceptions of food adequacy.

### Limitations and Strengths

 A limitation of this study is the absence of other important household-level SDoH factors, such as food stamp receipt, housing insecurity, and threats to utility services in the past 12 months, which should be assessed for a more comprehensive understanding of the home environment. Additionally, in this study, we assessed only the functional aspect of health literacy, respondents’ confidence in completing medical forms. While we interpret this measure in relation to related factors such as education and English proficiency, it is not a comprehensive measure of health literacy; and therefore, caution is warranted when interpreting the findings. Response bias, including social desirability and recall (memory) bias, may affect participants’ reports, particularly on sensitive topics such as food insecurity. We assume such biases are largely nondifferential between participants who agree and those who do not, meaning misreporting is expected to be broadly similar across these groups. Under this assumption, bias in measures of association is generally attenuated toward the null. We note, however, that this is a heuristic: recent and earlier work highlighted that nondifferential misclassification does not always bias results toward the null, especially in small samples or complex scenarios (Hamra, 2022; Yland et al., 2022). Nevertheless, for sufficiently large samples, this provides a reasonable and conservative framework for interpreting potential reporting bias. The cross-sectional design of this study also prevents the ability to assess causal relationships and may overlook long-term trends or dynamics in intra-household agreement on SDoH. Finally, another limitation lies in the interpretation of Kappa values, as proposed by Cohen (McHugh, 2012). While values ≤ 0 are considered to show no agreement and 0.41–0.60 as “moderate,” this may be misleading. It could imply that 40% disagreement is acceptable, though such a level of agreement is inadequate in fields like healthcare and clinical labs, where even 40% incorrect data may lead to harmful consequences. Many sources suggest 80% agreement as the minimum threshold for interrater reliability, and interpreting Kappa values below 0.60 as inadequate would be more appropriate for ensuring confidence in the results. In this context, our results fall well short of this threshold, with only two of the 19 items meeting it.

A strength of our study includes the use of independent reports from family respondents. The sample, comprising 277 households across Southern California, represented a reasonable size, sufficient: to be dropped to yield meaningful insights while acknowledging some limits in generalizability. Indeed, given the intentional focus on Latinos/Hispanics, the ability to draw broader comparisons to other ethnic or demographic groups is limited. Moreover, the sample was not designed to be representative of the broader Latino/Hispanic population in Southern California. The CDEs were also selected through a rigorous and thoughtful process that drew upon validated instrument libraries, cutting-edge research, and expert input from numerous nationally recognized specialists in the field.

### Public Health Implications and Research Perspectives

This study highlighted the limitations of relying on a single household member for reporting family-level SDoH, as it can introduce significant measurement error. However, involving multiple respondents may also lead to inconsistencies. Intra-household discrepancies could skew the identification of factors influencing health or health behaviors. This has important implications for the development, implementation, and effectiveness of preventive interventions, as interventions designed based on biased data may not accurately address the true needs of the population. To improve data accuracy, public health and research efforts might consider alternative strategies, such as encouraging joint responses, offering training on survey concepts, or supplementing self-reports with contextual data like geocoded indices. Additionally, selecting respondents based on criteria aligned with study goals (such as race/ethnicity or sex) may enhance data quality. Future research could also use mixed methods to better understand intra-household differences and similarities in SDoH reporting. Survey question phrasing (Bardasi et al., 2011; Davern et al., 2005) and structure (Koleck et al., 2024) can greatly influence SDoH responses, highlighting the need for instruments with strong psychometric properties across diverse communities. Clearly phrasing, and also explaining, questions seems crucial: interestingly, although living in the same household was a prerequisite for inclusion in this analysis, some respondents still reported living alone through the CDE questionnaire. This discrepancy may reflect differences in how the question is interpreted by various family members. For instance, some respondents might consider themselves as living “alone” if they occupy a separate part of the home, have limited day-to-day interaction with other household members, or manage their own finances independently. Research could also benefit from examining differences between child and parent perceptions of SDoH (Landry et al., 2019), especially food insecurity, where children as young as 6 may report more accurately than adults (Fram et al., 2015). Though our study was prevention-oriented, findings have clinical relevance and future research could explore caregiver agreement in these contexts. Food insecurity provides a clear example: by identifying families who are food insecure or at risk, healthcare providers can tailor care to address patients’ needs and resources while connecting them with relevant support, such as food assistance programs, and providing direct help when necessary. These efforts can improve overall health and reduce individual and community-level food insecurity, thus lowering healthcare costs by preventing unnecessary emergency visits and consultations (Eldred & Kameg, 2021; Healthcare Without Harm, 2018). Professional organizations, including the American Academy of Pediatrics, recommend SDoH screening, but adoption and implementation vary widely across clinical settings, potentially exacerbating health inequities: a study of pediatricians’ screening and referral practices in low-income families found that although most pediatricians (61.6%) thought that screening is important, fewer (39.9%) reported that screening is feasible or felt prepared addressing families’ social needs (20.2%) and screening tended to focus on childcare and transportation, with less attention to housing, food, or utilities insecurity (Garg et al., 2019).

## Conclusion

Our study examined the agreement between family member reports of household-level SDoH in Southern California and found discrepancies, with stronger agreement on demographics and transportation matters. Agreement on food insecurity items varied by dyad composition: same-sex dyads demonstrated greater agreement than mixed-sex dyads, whereas dyads in which both members identified as Latino/Hispanic showed lower levels of agreement. These findings seem to underscore the importance of tailored data collection strategies to enhance the accuracy and representativeness of household-level data, such as promoting joint responses and accounting for individual-level SDoH during assessment. Researchers and public health professionals might account for these variations to improve the accuracy of SDoH assessments and better address the needs of vulnerable populations.

## Supplementary Information

Below is the link to the electronic supplementary material.

ESM 1 DOCX (628 KB)

## Data Availability

The combined datasets used and/or analyzed during the current study are available from the corresponding author on reasonable request.
