# Supplementary material for: Evaluating Intra-household Agreement on Multi-domain Family-Level Social Determinants of Health and Exploring Individual Correlates of Agreement in Two Southern California Family Studies
Source: Prev Sci. 2026 May 12;27(4):571–81. doi: 10.1007/s11121-026-01914-2 (PMC13198477; doi:10.1007/s11121-026-01914-2)
Supplement: Supplementary file 1 — DOCX (628 KB) [file 11121_2026_1914_MOESM1_ESM.docx]

## Supplemental materials

### Supplemental Material 1. Selected Family-Level Common Data Elements (Self-Reported Social Determinants of Health).

| **Demographics** | | |
| --- | --- | --- |
| *Number of Family Members* | How many people currently live in the household? | 1-20; -77 Don't know; -88 Prefer not to answer |
| *Living Situation* | Which of the following best describes your current living situation? (Select ONE only) | 1 Live alone in my own home (house, apartment, condo, trailer, etc.); may have a pet 2 Live in a household with other people  3 Live in a residential facility where meals and household help are routinely provided by paid sta# (or could be if requested) 4 Live in a facility such as a nursing home which provides meals and 24-hour nursing care 5 Temporarily staying with a relative or friend 6 Temporarily staying in a shelter or homeless 90 Other -88 Prefer not to answer |
| **Economics** | | |
| *Household Income* | What is your best estimate of the total income of all family members from all sources, before taxes, in the last calendar year? [Based on 2022 Federal Poverty Guidelines] | 1, less than $13,590 \| 2, $13,590 to $18,309 \| 3, $18,310 to $23,029 \| 4, $23,030 to $27,749 \| 5, $27,750 to $32,469 \| 6, $32,470 to $37,189 \| 7, $37,190 to $41,909 \| 8, $41,910 to $46,629 \| 9, $46,630 to $51,349 \| 10, $51,350 to $56,069 \| 11, $56,070 to $60,789 \| 12, $60,790 to $65,509 \| 13, $65,510 to $70,229 \| 14, $70,230 to $74,949 \| 15, $74,950 to $79,669 \| 16, $79,670 to $84,389 \| 17, $84,390 to $89,109 \| 18, $89,110 to $93,829 \| 19, $93,830 to $98,549 \| 20, $98,550 to $103,269 \| 21, more than $103,269 \| -77, Don't know \|  -88, Prefer not to answer |
| *Financial Adversities (7)* | In the past 3 months, did you have trouble paying for any of the following?  Food \| Housing \|  Heat and electricity \|  Medical needs \|  Transportation \|  Childcare \|  Debts | 1 Yes; 0 No; -77 Don't know; -88 Prefer not to answer |
| ***Health and Medical Care*** | | |
| *Ever Delayed Medical Care for Financial Reasons* | During the past 12 months, have you DELAYED getting medical care because of the cost? | 1 Yes; 0 No; -77 Don't know; -88 Prefer not to answer |
| ***Housing*** | | |
| *Food Insecurity (5)* |  |  |
|  | "The food that (I/we) bought just didn't last, and (I/we) didn't have money to get more." Was that often, sometimes, or never true for (you/your household) in the last 12 months? | 1 Often true; 2 Sometimes true; 3 Never true; -77 Don't know -88 Prefer not to answer |
|  | "(I/we) couldn't afford to eat balanced meals." Was that often, sometimes, or never true for (you/your household) in the last 12 months? | 1 Often true; 2 Sometimes true; 3 Never true; -77 Don't know;  -88 Prefer not to answer |
|  | In the last 12 months, did you or other adults in your household ever cut the size of your meals or skip meals because there wasn't enough money for food? | 1 Yes; 0 No; -77 Don't know; -88 Prefer not to answer |
|  | In the last 12 months, did you ever eat less than you felt you should because there wasn't enough money to buy food? | 1 Yes; 0 No; -77 Don't know; -88 Prefer not to answer |
|  | In the last 12 months, were you ever hungry but didn't eat because you couldn't afford enough food? | 1 Yes; 0 No; -77 Don't know; -88 Prefer not to answer |
| *Lack of Transportation Impact (3)* |  |  |
|  | Has lack of transportation… Kept you from medical appointments? Kept you from doing things needed for daily living? Been a problem for you? | 1 Yes; 0 No; -88 Prefer not to answer |

### Supplemental Material 2. Distribution of Individual- and Household-Level Social Determinants of Health According to the Respondent Considered in the FRESH-LC Study (n=134 households).

|  | **Respondent 1 (n=134)** | **Respondent 2 (n=134)** |
| --- | --- | --- |
|  | **Frequency (%) or Mean (SD)** | **Frequency (%) or Mean (SD)** |
| **Individual level** |  |  |
| ***Demographics*** |  |  |
| *Sex (Female)* | 124 (92.5) | 31 (23.1) |
| *Ethnicity (Latino/Hispanic)* | 129 (96.3) | 120 (89.6) |
| *Age (Years)* | 39.8 (5.70) | 41.6 (10.1) |
| *Birthplace (Outside the United States)* | 72 (53.7) | 77 (57.5) |
| ***Economics*** |  |  |
| *Employment Status (Unemployed)* | 41 (30.6) | 28 (20.9) |
| ***Health and Medical Care*** |  |  |
| *Health Literacy (Not Excellent)* | 45 (33.6) | 88 (65.7) |
| **Household level** |  |  |
| ***Demographics*** |  |  |
| *Number of Family Members (3)* | 21 (15.7) | 16 (11.9) |
| *Living Situation (Alone)* | 61 (45.5) | 40 (29.9) |
| ***Economics*** |  |  |
| *Income (>more than $103.269/per year)* | 26 (19.4) | 29 (21.6) |
| *Financial Adversities* |  |  |
| Trouble Paying Medical (Yes) | 10 (7.5) | 8 (6) |
| Trouble Paying Food (Yes) | 13 (9.7) | 10 (7.5) |
| Trouble Paying Electricity (Yes) | 18 (13.4) | 13 (9.7) |
| Trouble Paying Housing (Yes) | 17 (12.7) | 20 (14.9) |
| Trouble Paying Transport (Yes) | 7 (5.2) | 6 (4.5) |
| Trouble Paying Debts (Yes) | 38 (28.4) | 27 (20.1) |
| Trouble Paying Childcare (Yes) | 6 (4.5) | 2 (1.5) |
| ***Health and Medical Care*** |  |  |
| *Ever Delayed Medical Care for Financial Reasons (Yes)* | 30 (22.4) | 26 (19.4) |
| ***Housing*** |  |  |
| *Lack of Transportation Impact* |  |  |
| Missed Medical Appointment (Yes) | 2 (1.5) | 4 (3) |
| Daily Life Inconvenience (Yes) | 4 (3) | 5 (3.7) |
| Problem For Me (Yes) | 128 (95.5) | 122 (91) |
| *Food Insecurity* |  |  |
| Cut Size or Skip Meals (Yes) | 9 (6.7) | 10 (7.5) |
| Eat less than You Felt (Yes) | 4 (3) | 6 (4.5) |
| Could Not Afford Balanced Meal (Often True) | 5 (3.7) | 4 (3) |
| No Money to Get More Food (Often True) | 1 (0.8) | 2 (1.5) |
| Hungry But Didn't Eat (Yes) | 7 (5.2) | 7 (5.2) |

SD: standard deviation

### Supplemental Material 3. Distribution of Individual- and Household-Level Social Determinants of Health According to the Respondent Considered in the FoodRx Study (n=143 households).

|  | **Respondent 1 (n=143)** | **Respondent 2 (n=143)** |
| --- | --- | --- |
|  | **Frequency (%) or Mean (SD)** | **Frequency (%) or Mean (SD)** |
| **Individual level** |  |  |
| ***Demographics*** |  |  |
| *Sex (Female)* | 102 (71.3) | 55 (38.5) |
| *Ethnicity (Latino/Hispanic)* | 140 (97.9) | 127 (88.8) |
| *Age (Years)* | 42.5 (7.13) | 43.1 (10.5) |
| *Birthplace (Outside the United States)* | 37 (25.9) | 40 (28) |
| ***Economics*** |  |  |
| *Employment Status (Unemployed)* | 11 (7.7) | 19 (13.3) |
| ***Health and Medical Care*** |  |  |
| *Health Literacy (Not Excellent)* | 28 (19.6) | 44 (30.8) |
| **Household level** |  |  |
| ***Demographics*** |  |  |
| *Number of Family Members (3)* | 49 (34.3) | 49 (34.3) |
| *Living Situation (Alone)* | 13 (9.1) | 5 (3.5) |
| ***Economics*** |  |  |
| *Income (>more than $103.269/per year)* | 70 (49) | 65 (45.5) |
| *Financial Adversities* |  |  |
| Trouble Paying Medical (Yes) | 5 (3.5) | 6 (4.2) |
| Trouble Paying Food (Yes) | 11 (7.7) | 9 (6.3) |
| Trouble Paying Electricity (Yes) | 15 (10.5) | 9 (6.3) |
| Trouble Paying Housing (Yes) | 15 (10.5) | 9 (6.3) |
| Trouble Paying Transport (Yes) | 6 (4.2) | 10 (7) |
| Trouble Paying Debts (Yes) | 30 (21) | 23 (16.1) |
| Trouble Paying Childcare (Yes) | 8 (5.6) | 4 (2.8) |
| ***Health and Medical Care*** |  |  |
| *Ever Delayed Medical Care for Financial Reasons (Yes)* | 7 (4.9) | 5 (3.5) |
| ***Housing*** |  |  |
| *Lack of Transportation Impact* |  |  |
| Missed Medical Appointment (Yes) | 1 (0.7) | 1 (0.7) |
| Daily Life Inconvenience (Yes) | 4 (2.8) | 4 (2.8) |
| Problem For Me (Yes) | 4 (2.8) | 6 (4.2) |
| *Food Insecurity* |  |  |
| Cut Size or Skip Meals (Yes) | 10 (7) | 7 (4.9) |
| Eat less than You Felt (Yes) | 5 (3.5) | 4 (2.8) |
| Could Not Afford Balanced Meal (Often True) | 8 (5.6) | 3 (2.1) |
| No Money to Get More Food (Often True) | 3 (2.1) | 2 (1.4) |
| Hungry But Didn't Eat (Yes) | 2 (1.4) | 7 (4.9) |

SD: standard deviation

### Supplemental Material 4. Self-Reported Household-Level Social Determinants of Health Agreement Assessment, Using Only Dyads That Jointly Self-Declared Living With Others (n= 174 households).


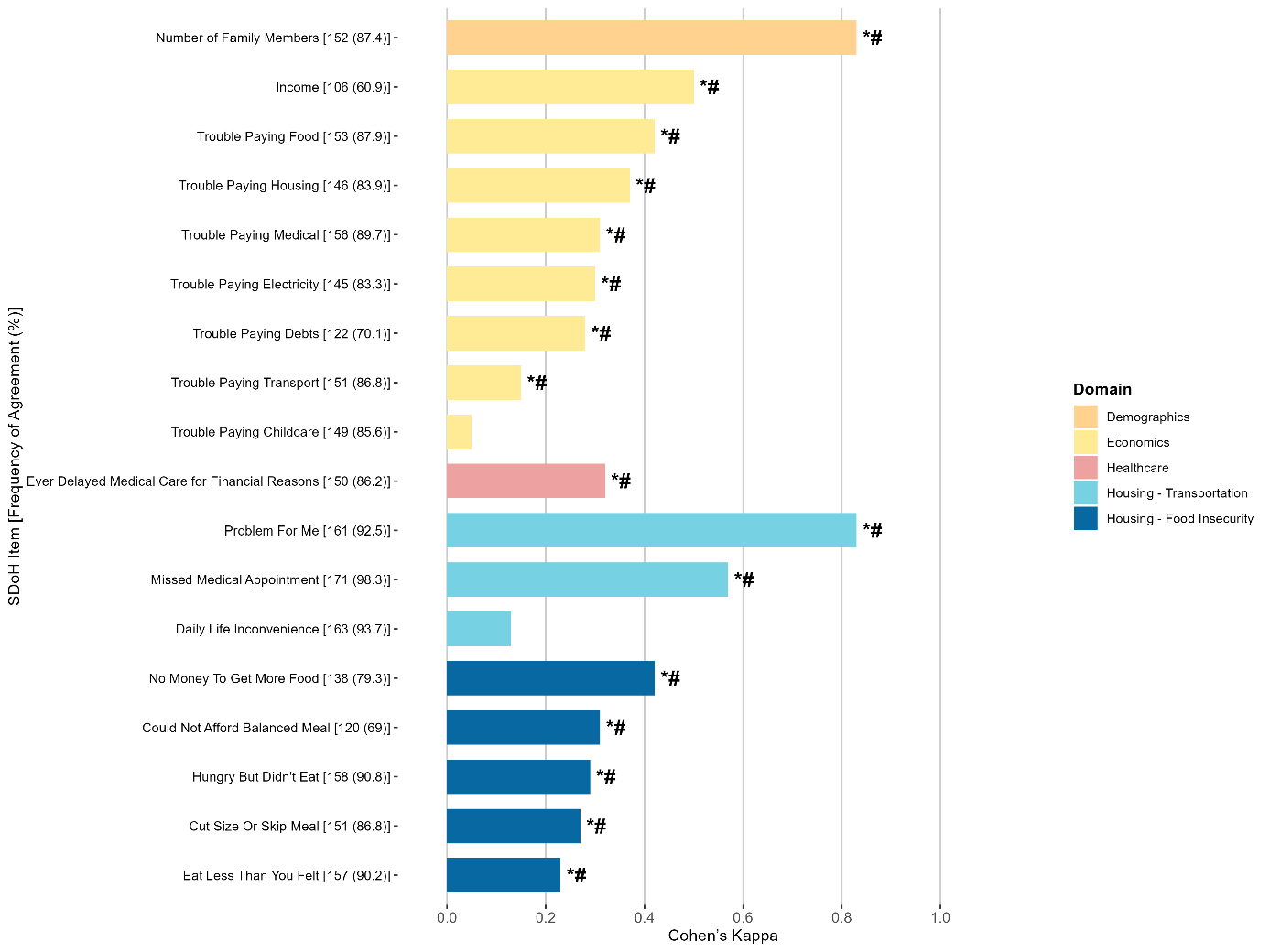


SDoH: Social Determinants of Health

For each (sub)-domain, variables are displayed in decreasing order of Cohen's Kappa. Cohen’s Kappa go beyond simply calculating the percentage of items agreed upon by raters; it accounts for the possibility of chance agreement. Its values range from 0 to 1, with 0 indicating no agreement and 1 indicating perfect agreement between the raters. Cohen’s Kappa can also be negative (disagreement).

*** p<0.05 (Kappa test, non-adjusted p-values); # p<0.05 (after adjusting for multiple comparisons)**

### Supplemental Material 5. Self-Reported Household-Level Social Determinants of Health Agreement Assessment, the FRESH-LC Study (n= 134 households).


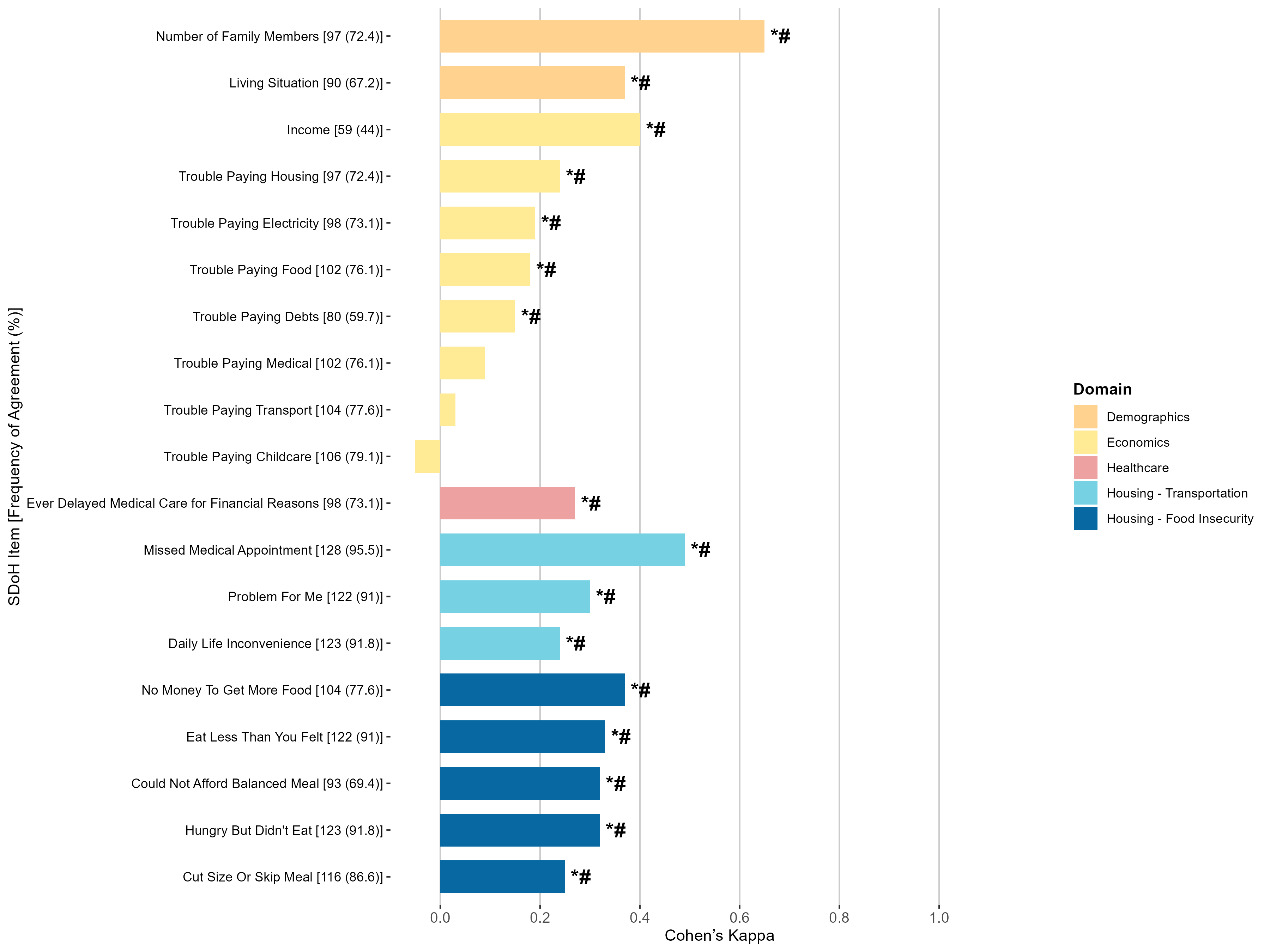


SDoH: Social Determinants of Health

For each (sub)-domain, variables are displayed in decreasing order of Cohen's Kappa. Cohen’s Kappa go beyond simply calculating the percentage of items agreed upon by raters; it accounts for the possibility of chance agreement. Its values range from 0 to 1, with 0 indicating no agreement and 1 indicating perfect agreement between the raters. Cohen’s Kappa can also be negative (disagreement).

*** p<0.05 (Kappa test, non-adjusted p-values); # p<0.05 (after adjusting for multiple comparisons)**

### Supplemental Material 6. Self-Reported Household-Level Social Determinants of Health Agreement Assessment, the FoodRx Study (n=143 households).


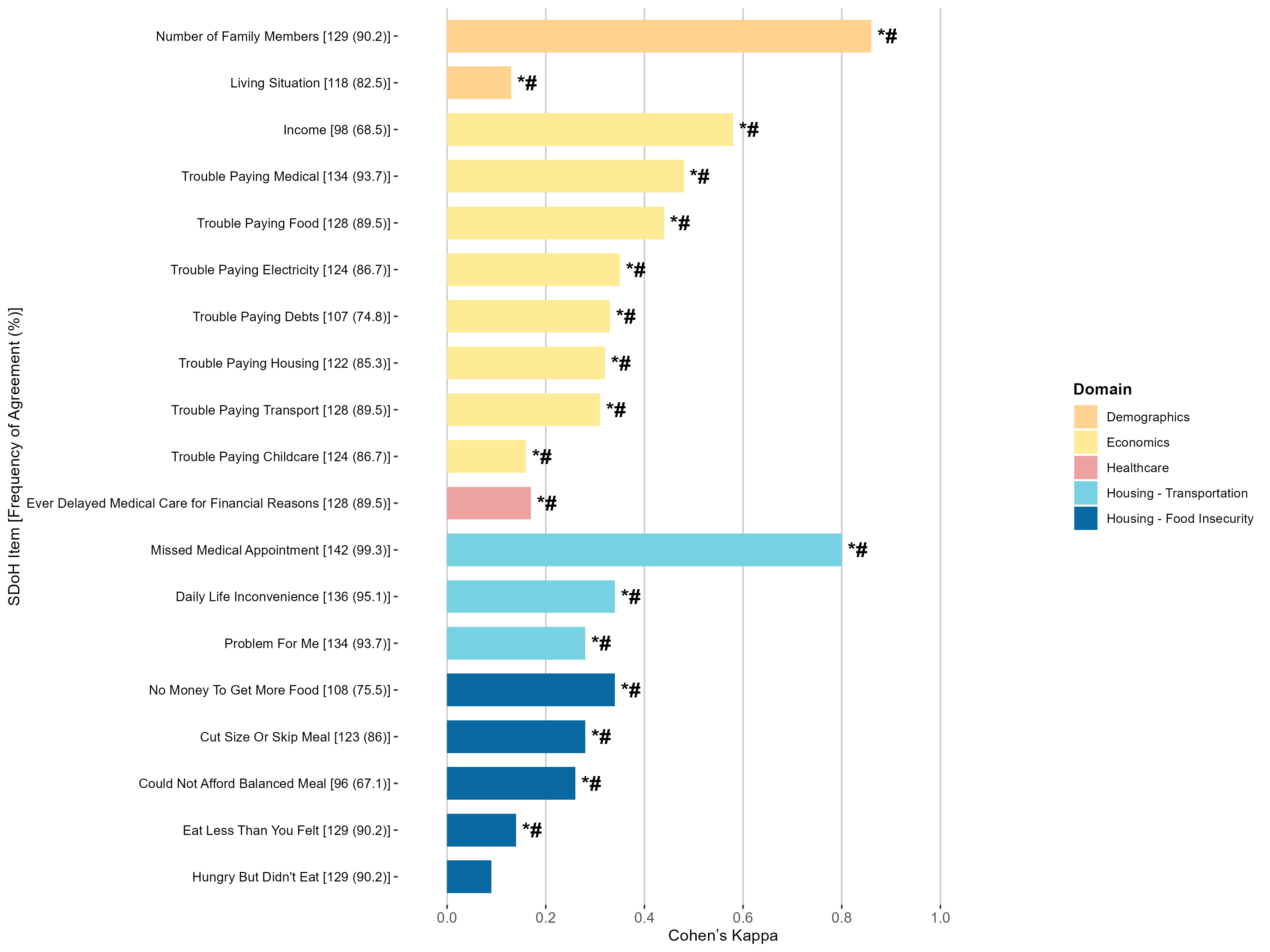


SDoH: Social Determinants of Health

For each (sub)domain, variables are displayed in decreasing order of Cohen's Kappa. Cohen’s Kappa go beyond simply calculating the percentage of items agreed upon by raters; it accounts for the possibility of chance agreement. Its values range from 0 to 1, with 0 indicating no agreement and 1 indicating perfect agreement between the raters. Cohen’s Kappa can also be negative (disagreement).

*** p<0.05 (Kappa test, non-adjusted p-values); # p<0.05 (after adjusting for multiple comparisons)**
